# Extraction, purification, kinetic and thermodynamic properties of urease from germinating *Pisum Sativum L.* seeds

**DOI:** 10.1186/1471-2091-15-15

**Published:** 2014-07-28

**Authors:** Mohamed E EL-Hefnawy, Mohamed Sakran, Ali I Ismail, Eman Fahmy Aboelfetoh

**Affiliations:** 1 Department of Chemistry, Rabigh College of Science and Arts, King Abdulaziz University, P.O. Box 344, Rabigh 21911, Saudi Arabia; 2 Department of Biochemistry, Faculty of Science, Tabuk University, Tabuk, Saudi Arabia; 3 Department of Chemistry, Faculty of Science, Tanta University, Tanta 31527, Egypt

**Keywords:** Urease, Enzyme activity, Enzyme purification, *Pisum Sativum L*, Pea seeds

## Abstract

**Background:**

Urease, one of the highly efficient known enzymes, catalyzes the hydrolysis of urea into ammonia and carbon dioxide. The present study aimed to extract urease from pea seeds (*Pisum Sativum L).* The enzyme was then purified in three consequence steps: acetone precipitation, DEAE-cellulose ion-exchange chromatography, and gel filtration chromatography (Sephacryl S-200 column).

**Results:**

The purification fold was 12.85 with a yield of 40%. The molecular weight of the isolated urease was estimated by chromatography to be 269,000 Daltons. Maximum urease activity (190 U/g) was achieved at the optimum conditions of 40°C and pH of 7.5 after 5 min of incubation. The kinetic parameters, *K*_*m*_ and *V*_*max*_, were estimated by Lineweaver-Burk fits and found to be 500 mM and 333.3 U/g, respectively. The thermodynamic constants of activation, ΔH, *E*_*a*_, and ΔS, were determined using Arrhenius plot and found to be 21.20 kJ/mol, 23.7 kJ/mol, and 1.18 kJ/mol/K, respectively.

**Conclusions:**

Urease was purified from germinating Pisum Sativum L. seeds. The purification fold, yield, and molecular weight were determined. The effects of pH, concentration of enzyme, temperature, concentration of substrate, and storage period on urease activity were examined. This may provide an insight on the various aspects of the property of the enzyme. The significance of extracting urease from different sources could play a good role in understanding the metabolism of urea in plants.

## Background

Catalyzing the hydrolysis of urea into ammonia and carbon dioxide, ureases (urea amidohydrolases, EC 3.5.1.5) are a one of known highly efficient enzymes that belong to amidohydrolase and phosphotriesterase superfamily [1]. Several reports have been published on the extraction of urease from various bacteria [2-4], and plants [5-11]. The high molecular mass nickel-containing metalloenzyme [12] is believed to play an important role in the nitrogen transport cycle in plants [13]. In addition, the enzyme decomposes urea formed from arginase that is found in seed germination [14]. Urease is also important in human bodies due to the fact that many urinary tract and gastroduodenal diseases [15,16], including cancer [17,18], are related in some ways to this enzyme. The increased need in finding proper ways to remove urea from different environments brought great attractions in the biotechnology field [19]. Some of urease's applications include treatment of industrial waste [20], the industry of alcoholic beverages [21], use in haemodialysis [22,23], and its potential use in space missions as life supporter [24].

The plant and fungal ureases are homo-oligomeric with identical proteins repetition. On the other hand, the bacterial ureases are composed of complex repetitions of two or three subunits of different sizes [25]. The crystal structure of protein is often the key to its enzyme function. This configuration is governed by its primary structure and environment. Any environmental factor, that alters the shape of the enzyme or blocks the access to the active site in substrate, will affect enzyme activity. Such environmental factors include matrix salt concentration, pH, temperature, substrate concentration, activators, and inhibitors [26,27].

The purpose of this study is to extract, purify, and characterize urease from plant source (*pisum sativum L*). Also, the activity of the enzyme was evaluated based on the change of the environmental factors that carried out during the purification procedure. This may provide an insight on the various aspects of the property of the enzyme.

## Methods

Pisum *Sativum L*. seeds were obtained from faculty of agriculture, Kafr Elshaikh University, Kafrelshaikh city, Egypt. The seeds were soaked in distilled water for 6 hours, germinated in the dark at 22°C for 2, 4, 6, 8, 10, 12, 14 and 16 days. The germinated seeds were stored separately in deep freezer (−20°C) for further experimental purposes. Dextran polymer particles (Sephadex G-200), bovine serum albumin (BSA), standard proteins, and DEAE-cellulose were purchased from Sigma Chemicals Ltd., USA. All other chemicals used for this research were of analytical grade. All absorbance measurements were performed using Lambda 35 PerkinElmer UV/V is spectrometer.

### Urease extraction and purification

Unless mentioned otherwise, all of the following procedures were done at 4°C. Ten grams of germinated seeds of *pisum sativum* were pasted in a mortar and pestle and then suspended in 40 mL of 20% chilled acetone (−20°C). Occasional stirring for 3 h was required. Double layer cheese cloth was used for filtrating of the suspension. After 15 minutes of centrifuging of the filtrate, the supernatant was isolated and used as “crude extract”.

The urease assay was performed as described by Sharma *et al*. [28]. Enzyme extract (0.25 μL) was added to 10 mL of urea solution (0.4 g urea in 25 ml of phosphate buffer). One millilitre of the previous solution was added to each test tube containing 5 mL of Nessler^’^s reagent, and incubated at 40°C for 5 min. They were followed by the addition of 1.0 M HCl to terminate the reaction after specific time. Absorbance measurements were taken for the resulting solutions (at 405 nm). The estimation of urease was carried out using the standard curve of ammonium sulphate. One unit of urease activity is defined as “the amount of enzyme required to liberate 1.0 μM of NH_3_ from urea per minute at pH 7.5 and temperature 40°C” [29].

Proteins were determined according to Lowry *et al.*[30] using BSA as standard material. Different concentrations of BSA were prepared ranging from (0 to 25) μg/mL. The linear calibration curve was used to determine the concentration of protein in the assay and estimated for the original sample.

The enzyme was purified to homogeneity by the following successive steps which carried out at 4°C:

### Acetone precipitation

The “crude extract” was adjusted to 50% saturation by addition of acetone (chilled to −20°C) under constant and gentle stirring. The resulting precipitate was centrifuged, collected, dissolved in minimum volume of pre-cold 50 mM phosphate buffer (pH = 7.4), and finally dialyzed against the same buffer for 24 h. The resulting solution was then centrifuged for 10 min and the clear supernatant was designated as “crude enzyme solution”.

### DEAE-cellulose chromatography

The “crude enzyme solution” was dialyzed against 50 mM phosphate buffer, pH 7.4. It was then loaded on pre-equilibrated DEAE-cellulose column (15 × 3.0 cm) (with 50 mM phosphate buffer, pH 7.8). The bound proteins were eluted with a linear gradient of NaCl (100 – 500 mM), prepared with phosphate buffer, pH 7.8, at a flow rate of 0.5 mL min^−1^. After collecting the active fractions (the fractions that shows urease activity), the proteins binding to the column were eluted using gradient of (0–0.5 M) KCl and (20 mM) phosphate buffer, pH 7.5. The absorbance of these fractions was measured at wavelength of 280 nm. The active fractions were combined and the volume was measured for the determination of the urease activity and protein contents in the assay.

### Gel filtration chromatography

The enzyme obtained from the ion exchange step was concentrated with acetone and loaded on the Sephacryl S-200 column (1.5 × 65) at a flow rate 30 mL/h using 50 mM phosphate buffer, pH = 7.8. Five millilitre eleunts were collected. The enzymatically active fractions were pooled and dialyzed against 50 mM phosphate buffer, pH 7.4 for 24 h. The absorbance measurements at 280 nm were used to determine the protein concentration and urease activity.

### Enzyme characterization

The isolated enzyme activity was characterized and studied as a function of pH, temperature, storage period, enzyme concentration, and substrate concentration using the following procedures:

### Determination of molecular weight of urease

Two millilitres of blue dextran-2000 solution (6 mg into 3 mL of PEM buffer pH 7.5, 0.1 M PIPES, 1 mM EGTA, 1 mM MgSO4, at pH = 6.6; where PIPES is piperazine-N, N′-bis(2-ethanesulfonic acid, and EGTA is ethylene glycol-bis-(β-aminoethyl ether)-N,N,N',N'-tetraacetic acid) were passed through Sephacryl S-200 column. 20 mM of PEM buffer pH 7.5 was added. Fractions of 5 ml were eluted and the absorbance at 600 nm for each fraction was measured. The column void volume (*V*_*0*_) was determined by estimating the total volume of the fractions characterized with the starting point movement of the dextran to climax of absorbance of the blue dextran. Same procedure was done for the standard proteins; BSA, aldolase, catalase, ferrtin, and thyroglobulin. The eluted fractions, which give a maximum absorbance at 280 nm, were determined, and the eluted volume (*V*_*e*_) was calculated for each standard protein. The linear calibration curve of $\frac{V_{e}}{V_{0}}$ against logarithm value of molecular weight of standard protein was plotted. The curve was used for determining the molecular weight of native urease.

### Effect of pH on the activity of *pisum sativum* urease

The pH profile for the purified urease was estimated using urea as a substrate. The pH range used was from 3 to 10 using 50 mM phosphate buffer.

### Effect of storage at −4°C on enzyme activity

To determine the effect of storage of the enzyme on the urease activity, the enzyme was stored at different time internals of 0–60 days. The enzyme activity was measured after each separate time period.

### Effect of different concentration of enzymes

The optimum enzyme concentration was determined by varying the amount of the pure enzyme.

### Effect of temperature

The optimum temperature for urease activity was determined over the temperatures from 10 to 40°C using the standard conditions of the assay.

### Thermodynamic studies

The relationship between the rate of an enzymatic reaction and activation energy is given by the empirical formula of the Arrhenius equation:

(1) $$E_{a}=\;R\cdot \ln\left(\frac{V_{2}}{V_{1}}\right)\cdot \left(\frac{1}{T_{1}}‒\frac{1}{T_{2}}\right)$$

where *V*_*1*_ and *V*_*2*_ are the enzyme activities at the temperatures *T*_*1*_ and *T*_*2*_; *E*_*a*_ is the energy of activation (kJ mol^−1^) which can be determined from the slope of the Arrhenius plot of ln(*V*) against $\frac{1}{T}$.

The activation enthalpy (ΔH) can be calculated by eqn. 2.

(2) $$\Delta H=E_{a}-\mathrm{RT}$$

Finally the entropy (ΔS) was calculated by eqn 3 (Eyring-Polanyi), which correlates ∆H, *E*_*a*_, and Arrhenius equation (eqn. 1);

(3) $$\ln\left(\frac{V_{\text{max}}}{T}\right)=\ln\left(\frac{K_{B}}{h}\right)+\frac{\mathrm{\Delta S}}{R}-\frac{\mathrm{\Delta H}}{R}\cdot \frac{1}{T}$$

where T, *K*_*B*_, h, and R are absolute temperature, Boltzmann constant, Planck constant and gas constant respectively.

### Effect of different concentration of substrates

The effect of urea concentration on the activity of enzyme was examined. Urea solution of different concentration was taken in different test tubes and the enzyme activity was measured. *K*_*m*_ and *V*_*max*_ for urease were calculated using Lineweaver-Burk double reciprocal plot [31].

## Results

### Purification of urease

After 6 days of germination the activity increased gradually and showed maximum activity on the ninth day after germination and then declined rapidly (Data not shown). Therefore, we used the 9^th^ day of germination for further experimental purpose. The results of the purification activity of the germinated *pisum sativum* seed urease were summarized in Table 1. The total activity which represents the summation of the activity of all proteins in the extract samples showed a decrease over the period of the purification procedure. However, the most important parameter is the specific activity of the extracted enzyme which represents the actual activity of the active proteins only. The results showed an increase in the specific activity throughout the purification steps. The final purification fold achieved was nearly 12.85. The specific activity after finishing the purification was 5833.3 Umg^−1^.

**Table 1:** **Yield and purification fold at different steps of purification of urease from germinated *pisum sativum *** **seeds**

**Step of purification**	**Total protein (mg/ml)**	**Total activity (Units)**	**Specific activity (U/mg)**	**Yield (%)**	**Purification fold**
Crude extract	22	10000	454.5	100	1
Acetone precipitation and dialysis	12	7500	625	75	1.3
Ion exchange DEAE-cellulose	3.1	5000	1613	50	3.5
Gel filtration Sephacryl S-200	0.72	4200	5833	42	12.85

**Figure 1:**
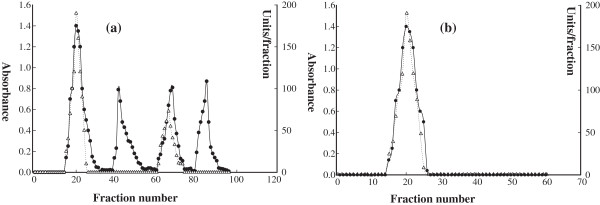
**A typical elution profile for the ion exchange chromatography of *Pisum Sativum L. *** **urease from, (a) only DEAE-cellulose column (15 × 3.0 cm), and (b) DEAE-Cellulose followed by Sephacryl S-200 column (90 × 1.6 cm i.d.). is for absorbance at 280 nm, and is for urease activity.**

**Figure 2:**
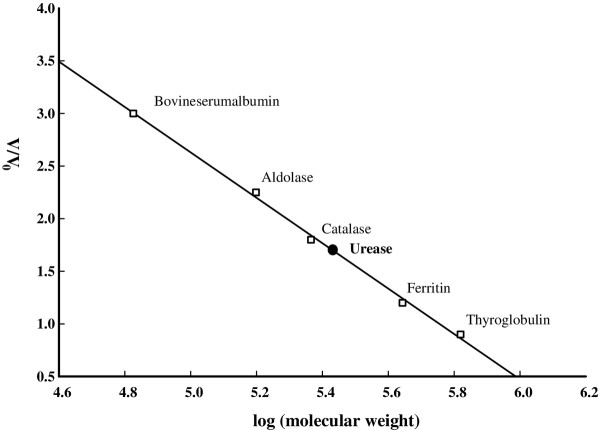
**Calibration curve for standard proteins; bovineserum albumin, aldolase, catalase, ferritin, and thyroglobulin using Sephacryl S-200 column.** The empty filled squares are for the standard proteins, and the solid sphere is for the urease extracted from the germinating pisum sativum seeds used in this study.

Ion-exchange chromatography was carried out using the anion exchanger DEAE-cellulose. Approximately 20 ml of crude extract was passed through the column. As shown in Figure 1a, four peaks of proteins appeared at fractions of 20, 41, 68, and 85. Peaks of fraction number 20 and 68 showed urease enzyme activity. The values of the urease activity were of 190 unit/ fraction for the fraction number 20 and 73 unit/ fraction for the fraction number 68. The fractions of the first peak were collected for the gel chromatography separation. The fractions that showed enzyme activity from the gel chromatography filtration were concentrated by dialysis and applied to Sephacryl S-200 column. At the end of the purification procedure, one peak for protein and enzyme activity was observed at fraction 23 (Figure 2b).

### Characterization of the purified Urease

#### Molecular weight determination

The molecular weight of urease was determined using gel filtration chromatography to be equal to 269,000 ± 200 Da (linearity regression parameter of 5 standard proteins, R^2^ = 0.997) as shown in Figure 2.

### Effect of pH on the activity of *pisum sativum* urease

**Figure 3:**
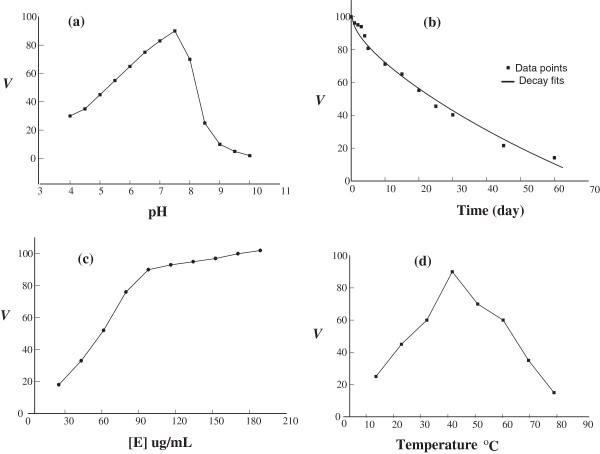
**Effect of (a) pH (at incubation temperature = 40°C), (b) time of storage (at −4°C), (c) enzyme concentration (pH = 7.5, temperature = 40°C), and (d) temperature (pH = 7.5), on urease activity extracted from *Pisum Sativum L. *** **seeds.**

The pH profiles for the purified urease were estimated using urea as a substrate. The pH range used was from 3 to 10 using 50 mM phosphate buffer. The activity of urease was the highest at pH 7.5 (Figure 3a).

### Effect of storage period

The enzyme activity decreased with time even when stored at −4°C. It represented 100% on the first day but decreased to 80% on the tenth day. However, it retained about 14.1% even after 2 months. The exponential decay fits showed a half life time of 22.4 days (see Figure 3b).

### Effect of enzyme concentration on the activity of *pisum sativum* urease

As indicated in Figure 3c, the urease activity increased significantly rapidly by increasing the enzyme concentration until reaches a value of 100 ug/mL. After that the activity kept rising but in slower rate. The maximum activity reached was 102 units/ assay at 200 ug/mL of enzyme.

### Effect of temperature on enzyme activity

Figure 3d showed the temperature optimum curve for urease. The complete assays of enzyme were incubated at different temperatures from 10 to 80°C for 10 minute. Results showed that urease had an optimum temperature at 40°C.

### Thermodynamic parameters (E_a_, ∆H, and ∆S)

**Figure 4:**
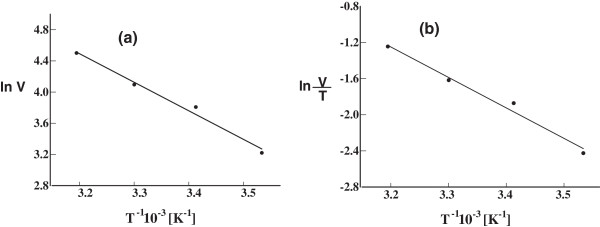
**Arrhenius plot for the activity of urease that is extracted from germinating *pisum sativum L. *** **seeds, for the calculation of (a) activation energy, and (b) entropy of activation and enthalpy of activation.**

The activation energy (E_a_) can be determined from the slope of the empirical formula of the Arrhenious plot of natural logarithm of the urease activity versus the reciprocal value of temperature (see Figure 4a) The activation energy was found to be 23.7 kJ/mol. Both enthalpy of activation (∆H) and entropy of activation (∆S) were calculated using Arrhenius plot as shown in Figure 4b and were found to be 21.20 kJ/mol, 1.18 kJ/mol respectively.

### Effect of substrate concentration on the activity of *pisum sativum* urease

**Figure 5:**
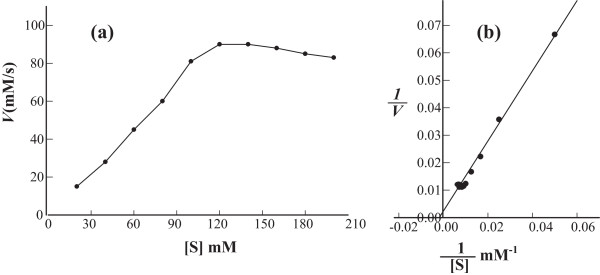
**Effect of substrate concentration on the activity of urease extracted from pisum sativum L. seeds. (a)** Dependence of initial reaction rate on substrate concentration of urease; pH 7.5, temperature 40°C. **(b)** Lineweaver-Burk double reciprocal plot.

As indicated in Figure 5a by increasing urea concentration, the activity increased until nearly constant maximum activity 102 units/assay at 200 mM of substrate. Further increase in urea concentration resulted in a gradual decrease in enzyme activity.

### The kinetics constants (*K*_m_ and *V*_max_)

The kinetics constants (*K*_*m*_ and *V*_*max*_) for the purified urease were determined by incubating a fixed amount of enzyme with varying the concentration of urea solution (urea used here as substrate). *K*_m_ and *V*_max_ for urea were calculated using Lineweaver-Burk double reciprocal plot, Figure 5b, and were found to be 500 mM and 333 U/g, respectively

## Discussion

Urease plays an essential role in the nitrogen metabolism in both germination and seedlings of plants. Two peaks were found to have urease activity. However, the highest activity peak was chosen for further experiments. The low activity peak (high salt peak) has low concentration and low urease ratio that limit our ability to consider it for further experimentation. The fact for having two peaks is unusual. However, as this study is the first to use the germinating pisum sativum seeds, there is a possibility of having more than one isozyme with different elution times and conditions. The results of germinated *pisum sativum* seed urease, Table 1, shows that the final purification fold achieved was 12.85 and the specific activity was 5833.3 Umg^−1^. In comparison with other studies, the purification results of germinated *chickpea* specific activity was 489.57 and the final purification fold was 45 [26]. Also, for *Proteus mirabilis urease*, the Specific activity of the extracted enzyme was 22932.86 and the final purification fold was 13.86 [32].

The molecular weight of the *pisum sativum* seeds urease reported in this investigation was 269,000 Da, compared to 480,000 Da for jack bean [33], and 540,000 Da for dehusked pigeonpea (*Cajanus cajan L*.) [7]. The molecular weight of enzymes is known to change relative to the source and even stage of plant growth.

The pH plays an important role in the activity of enzyme. The urease isolated from *Pisum Sativum* seed was found to yield maximum activity at pH 7.5 (Figure 3a) which means that the seeds may belong to the category of basic urease. Despite the fact that Mulberry leaves have shown neutral optimum pH [3], many other studies reported basic pH as an optimum value for the ectracted urease. For example the optimum pH was found to be 8 in jack beans [34], *pigeonpea*[7], pathogenic fungus (*Coccidiodes immitis)*[35], *aspergillus niger*[36], and *bacillus pasteurii*[37]. These results may be explained by the fact that acidic pH has an inhibitory effect on the enzyme resulting in reducing its activity. Also, the existence of the active sites in amino acids will be influenced by the change in pH which may alters the ionization of these amino acids [38].

The optimum temperature, where the greatest urease activity carries out, is equal to 40°C. This result is comparable to several studies, reported by Das *et al*. [7], and Srivastava *et al*. [39]. The kinetic energy of molecules increases with an increase in temperature which results in seeding up the rate of reaction. When the temperature was further increased, the molecules of enzyme exceed the barrier of energy. This causes the breakage of hydrogen and hydrophobic bonds that are responsible for maintaining the 3D structure of enzyme [4,40].

The optimum value of substrate concentration, where the urease activity has the largest value, was found to be 120 mM. After that the activity starts to gradually decrease. The decrease in the activity could be explained by substrate inhibition at higher urea concentrations. The enzyme showed the highest activity when incubated for 5 min under standard conditions; temperature = 40°C and pH = 7.4. The rate of hydrolysis of urea increases with increasing urea concentration until reaching a maximum, beyond that hydrolysis activity starts to decrease [41,42]. Loest [43] and Shepard and Lunceford [44] obtained maximum urease activity at 0.25 M and 0.008 M concentration of urea, respectively.

The Kinetics constants (*K*_*m*_ and *V*_*max*_) for urease extracted from germinated *pisum Sativum* was calculated using Lineweaver-Burk double reciprocal plot and were found to be 500 mM and 333.3 U/g respectively. These values indicated a low affinity of substrate to urease.

## Conclusions

Urease was purified from germinating Pisum Sativum L. seeds. The purification fold was 12.85 with a yield of 40%. The molecular weight was estimated as: 269,000 Daltons factors adjusted for its action was pH 7.5, temperature 40°C, but further studies are required to elucidate its significance in the metabolism of urea in plants.

## Abbreviations

DEAE: Diethylaminoethanol; BSA: Bovine serum albumin; PEM buffer: 0.1 M PIPES, 1 mM EGTA, and 1 mM MgSO4; PIPES: Piperazine-N,N′-bis(2-ethanesulfonic acid; EGTA: Ethylene glycol-bis-(β-aminoethyl ether)-N,N,N',N'-tetraacetic acid.

## Competing interests

The authors declare that they have no competing interests.

## Authors’ contributions

ME and MS conceived the study, carried the purification procedure, activity measurements, and drafted the manuscript. AI participated in the activity measurements, coordination, and helped drafting the manuscript. EA participated in the activity measurements, and coordination. All authors read and approved the final manuscript.
